# Self-efficacy, support and sustainability – a qualitative study of the experience of establishing breastfeeding for first-time Australian mothers following early discharge

**DOI:** 10.1186/s13006-020-00337-1

**Published:** 2020-11-23

**Authors:** Lucy James, Linda Sweet, Roslyn Donnellan-Fernandez

**Affiliations:** 1 College of Nursing and Health Sciences, Flinders University, Bedford Park, South Australia Australia; 2 School of Nursing and Midwifery, Deakin University, Burwood, Victoria Australia; 3 Centre for Quality and Patient Safety Research, Western Health Partnership, St Albans, Victoria Australia; 4 School of Nursing and Midwifery, Griffith University, Logan, Queensland Australia

**Keywords:** Breastfeeding, Early discharge, Postnatal care, First-time mothers, Breastfeeding support, Self-efficacy

## Abstract

**Background:**

Ensuring women receive optimal breastfeeding support is of key importance to the health of mothers and their infants. Early discharge within 24 h of birth is increasingly common across Australia, and the practice of postnatal home visiting varies between settings. The reduction in length of stay without expansion of home visits reduces midwives’ ability to support breastfeeding. The impact of early discharge on first-time mothers establishing breastfeeding was unknown. The study aim was to understand the experiences of first-time Australian mothers establishing breastfeeding when discharged from the hospital within 24 h of a normal vaginal birth.

**Methods:**

A qualitative interpretive method was used. Semi-structured interviews with 12 women following early discharge were conducted. Data were audio recorded, professionally transcribed, and subjected to a thematic analysis.

**Results:**

Three interconnected themes of ‘self-efficacy’, ‘support’ and ‘sustainability’ were identified. Self-efficacy influenced the women’s readiness and motivation to be discharged home early and played a role in how some of the mothers overcame breastfeeding challenges. Social, semi-professional and professional breastfeeding supports were key in women’s experiences. Sustainability referred to and describes what women valued in relation to continuation of their breastfeeding journey.

**Conclusion:**

This study found accessible people-based breastfeeding services in the community are valued following early discharge. Furthermore, there is demand for more evidence-based breastfeeding educational resources, potentially in the form of interactive applications or websites. Additionally, a focus on holistic and individualised breastfeeding assessment and care plans prior to discharge that link women with ongoing breastfeeding services is paramount.

## Background

Ensuring women receive optimal breastfeeding support is of key importance to the health of mothers and their infants. Breast milk is an unequalled, perfectly balanced source of nutrition for infants and is known to provide significant health benefits to mothers and babies [1, 2]. Furthermore, breastfeeding’s preventative factors for chronic diseases are an effective method of lowering health expenditure overtime by reducing long-term morbidity and mortality [1, 3].

The current global public health recommendations for infant feeding state infants should be exclusively breastfed for the first 6 months of life, and thereafter be introduced to other sources of food whilst continuing to breastfeed up to 2 years of age and beyond [1, 4]. However, Australia’s exclusive breastfeeding statistics fall well below these global recommendations [5]. In the most recent national report, Australia’s breastfeeding initiation rate was high at approximately 92% [5]. However, exclusive breastfeeding rates decline with 72.6% infants being exclusively breastfed at 2 months of age and 24.7% at 6 months of age [5]. No reasons for the decline were provided in this report.

In recent years, postnatal length of stay has reduced considerably across the globe including Australia [6, 7]. Although it varies within settings, the Australian median length of hospital stay for women who have a normal vaginal birth is 2 days [8]. In South Australia, current policy in public hospital settings promotes discharge within 24 h following an uncomplicated vaginal birth. However, this reduction in length of stay has not been accompanied by expansions to domiciliary home visiting services [9]. The World Health Organization recommend women stay in a health facility following birth for at least 24 h, and receive at least three postnatal visits, including home visits, in the first 6 weeks after birth [10]. The number of postnatal visits a woman receives in Australia is determined by the model of maternity care she has, and may vary from none to numerous visits during the first 6 weeks. For example, it is common for women in the public hospital system to receive one to two home visits in the first postnatal week; in private maternity hospitals domiciliary service is rare and hence women in this model of care receive no home visits; while women in midwifery continuity of care models receive as many home visits as needed over a six-week period.

There are two proposed reasons for the reduction in the length of postnatal stay; namely, reducing health expenditure, and improving women’s satisfaction [11, 12]. Significant health budget cuts have occurred in Australia in recent years, and reducing length of hospital stay is one way of lowering health costs [13]. The alternate justification is the improvement in women’s satisfaction. Early discharge intends to improve maternal satisfaction by offering advantages such as autonomy, increased sense of belonging, promoting a feeling of responsibility and participation, and facilitating family support in a comfortable home environment [11, 12, 14, 15]. However, it is unclear if early discharge meets the long-term outcomes such as breastfeeding continuation.

A literature review was conducted to explore the evidence on what women value in regard to breastfeeding initiation and support, and the impact early discharge may have on these values and practices [6]. The initial intent of this review was to examine literature defining early discharge as discharge within 24 h of birth. It became evident that there is no universal definition of ‘early discharge’. Due to inconsistent definitions of early discharge worldwide and minimal literature using the 24-h definition, research defining early discharge up to 72 h postpartum was used.

There were 21 articles published between 2005 and 2015 that met the inclusion criteria, and seven factors were identified which represent what women valued when initiating breastfeeding [6]. They were: trust and security [15–18], consistent advice [17, 19–25], practical/active breastfeeding support [17, 19–25], breastfeeding education [15, 16, 19, 23, 24, 26, 27], comfortable environment [7, 15, 17, 18, 23–25, 27–31], positive attitudes [15–20], and emotional support and individualised care [7, 17, 23, 27, 32]. The literature review highlighted the lack of research regarding the potential impact early discharge within 24 h may have on establishing breastfeeding, and in particular for first-time mothers [6].

The aim of this study was to understand the experience of first-time mothers establishing breastfeeding following early discharge. The research question was “What is the experience of first-time mothers establishing breastfeeding following early discharge within 24 hours of normal vaginal birth?”

## Methods

Interpretive descriptive approach [33] was used as it enables understanding of a phenomenon for the purpose of capturing subjective experiences and generating interpretations which have the ability to inform clinical practice.

### Ethics

Ethical approval for this study was granted by a university research ethics committee. All participants voluntarily agreed to participate and provided written informed consent. Anonymity is protected by the use of pseudonyms.

### Participants and recruitment

Twenty potential participants were identified through a different study about women’s infant feeding experiences, conducted on the social media site Facebook. Women who met this study’s inclusion/exclusion criteria (based on their responses and survey skip logic) were invited to self-nominate for this subsequent research. This study sought women who were primiparous, had a normal vaginal birth of a term infant, antenatally intended to breastfeed, were discharged within 24 h of birth and spoke English. The two studies were otherwise conducted independently. From the pool of 20 self-nominated potential participants, recruitment ceased at 12 as data saturation was reached.

### Data collection

**Fig. 1:**
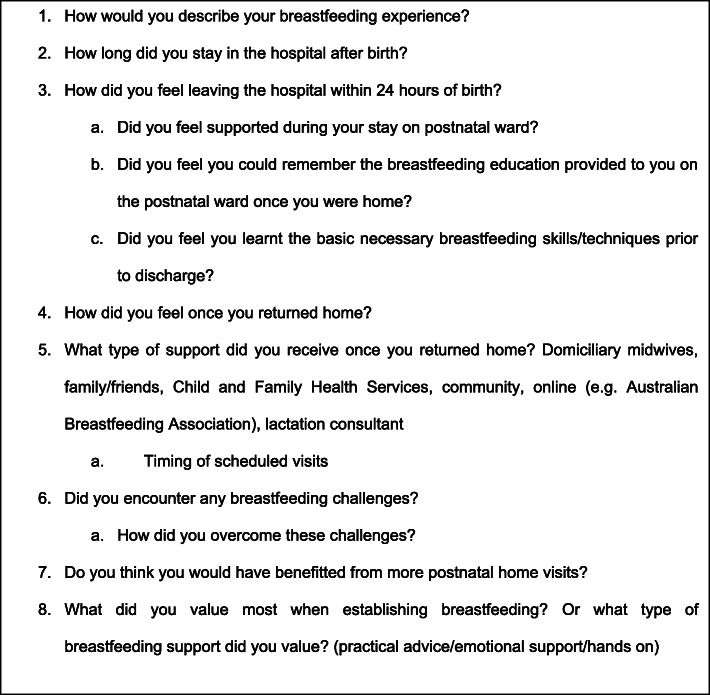
Interview guide

A semi-structured interview guide was created to elicit in-depth participant responses based on the literature review and professional experience. The interview schedule included eight open ended questions (and prompts) as shown in Fig. 1. The interview guide was piloted on a first-time mother who had recently given birth and was discharged home within 24 h of birth. The pilot interview was conducted to test participant experience of the interview guide and train the principal researcher. This data was not included in the final data corpus. Twelve interviews were conducted by the principal researcher using mediums/venues chosen by the participants; nine occurred over the telephone, one was conducted via Skype and two were conducted in person. Interviews lasted on average 45 min. All interviews were digitally audio recorded and transcribed verbatim by a professional secretariat. The principal researcher de-identified and checked all transcripts for accuracy.

### Data analysis

The dataset was analysed following the six-phase approach to thematic analysis as described by Braun and Clarke [34]. Thematic data analysis was performed simultaneously with data collection, allowing for recognition of data saturation. The initial coding of the data was conducted by the principal researcher. The two senior researchers undertook independent coding of a small subset of data to cross-check coding. The research team collectively reviewed coding and established the final themes.

## Results

**Fig. 2:**
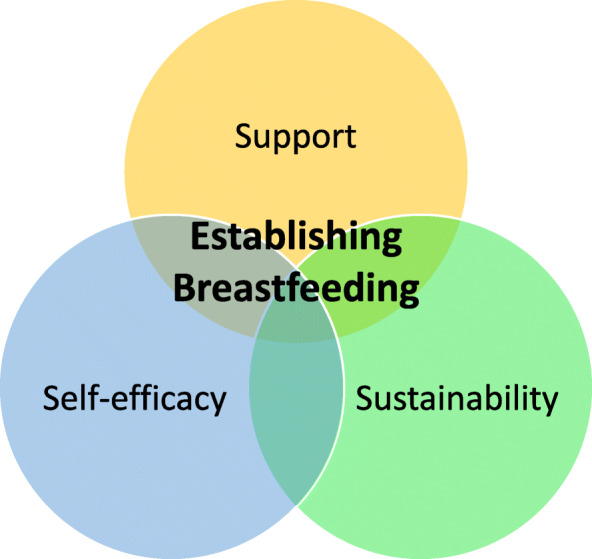
Venn diagram of themes

The demographic profile of the participants is shown in Table 1. Following data analysis, three interconnected themes were identified and labelled as ‘self-efficacy’, ‘support’ and ‘sustainability’, as shown in Fig. 2.

**Table 1:** Participant characteristics

Interview Number	Pseudonym	Age group	State of Australia	Model of care	Age of baby at time of interview
1	Betty	35 or more years old	New South Wales	Midwifery Group Practice	6 weeks
2	Cindy	25 to 29 years old	Queensland	Private midwife care	11 weeks
3	Sally	25 to 29 years old	South Australia	Regular hospital care	8 months
4	Nadia	25 to 29 years old	Queensland	Midwifery Group Practice	15 weeks
5	Anna	18 to 24 years old	South Australia	Regular hospital care	7.5 months
6	Lori	25 to 29 years old	South Australia	Private obstetric care	15 weeks
7	Melanie	25 to 29 years old	Northern Territory	General Practitioner shared care	15 Weeks
8	Christine	25 to 29 years old	Western Australia	Midwifery Group Practice	10 weeks
9	Diane	30 to 34 years old	Australian Capital Territory	Midwifery Group Practice	13 weeks
10	Josephine	18 to 24 years old	Northern Territory	Midwifery Group Practice	7 months
11	Ericka	18 to 24 years old	South Australia	Regular hospital care	7 months
12	Kate	30 to 34 years old	Victoria	General Practitioner shared care	5 months

### Self-efficacy

The participants described a sense of strength, determination and confidence in their ability to breastfeed following early discharge. These personal qualities are collectively known as one’s self-efficacy. Of the 12 women, all but one initially felt positive about discharging home within 24 h of birth. The women’s enthusiasm to be discharged early was motivated by their desire to commence their journey into motherhood and breastfeed in their own surroundings. Narelle said, “*It just was really lovely for me to be able to come home and settle straight back into our environment*”. Feeling at ease was important to these women, and many felt the hospital was not conducive with this. Cindy said, “*I guess the hospital doesn’t make me feel comfortable at all*.. . *I didn’t feel like I needed to be there. There wasn’t really any reason*”. For two women, their motivation to be discharged early was due to negative experiences with midwives on the postnatal ward. Anna felt distressed by the lack of support on the ward and felt it negatively impacted her confidence and ability to breastfeed. Being home positively influenced her breastfeeding experience and confidence: “*We really did struggle for the first few hours and then once we got home, she literally just figured it out*.. . *I think my feelings (of being relaxed) were projected onto her*”*.*

Cindy and Christine were both midwives and had strong determination to breastfeed. Cindy was passionate about discharging as early as possible to learn to breastfeed in her home environment, and about not giving formula to her baby. She said, “*For my husband and I, formula was never an option. Even if we had a sick baby in the nursery, we probably would have wanted IV [sic] drip or donor breastmilk, or anything like that before we went to formula. To us, breastfeeding is normal*”*.* Similarly, Christine was highly motivated to breastfeed, but concerned she may not breastfeed successfully having witnessed many challenges new mothers encountered through her work. Christine said, “*I could see how difficult breastfeeding is*... *I was worried that it wouldn’t happen for me or my milk wouldn’t come in*”. Although her confidence was initially lacking, Christine channelled these fears into motivation, stating “*Because I knew how hard it can be for women*... *I think I was more determined to make my breastfeeding work*”*.* These two women were driven by their strong intentions to breastfeed displayed a strong sense of self-efficacy, which assisted them to overcome challenges.

Kate’s intention was not to breastfeed at all, because she was embarrassed at the idea of feeding in public: “*I was adamant I was going to formula feed*”. After some skin-to-skin time with her baby and focused care by some motivated midwives prior to discharge, Kate realised she could breastfeed and that her baby loved breastfeeding, which filled her with confidence. She stated, “*Now I kind of enjoy breastfeeding more than I thought I would*”. This experience before her early discharge boosted her self-efficacy and influenced her intention to breastfeed.

Self-efficacy played a role in how some mothers overcame breastfeeding challenges in the early days and weeks of being home. Betty eagerly discharged home 6 hours after birth, but once home encountered issues with positioning and attachment. Unable to seek support from her midwifery group practice midwife, she returned to the hospital and was readmitted to the postnatal ward. Here Betty experienced moments of success which were key in overcoming her challenges with attachment and enhanced her confidence. She said, “*I’ve just got to do it and build my confidence*... *it’s me having the experience of it being right, to know when it’s right and when it’s not right*”. When she discharged home the second time, she felt “*okay now I know what I am doing, I’ve got confidence in this*”. Similarly, after discharging home, Nadia was challenged by low supply and extensive nipple pain with her baby slow to gain weight. Through perseverance she was successfully breastfeeding without shields, and pain free at three and a half months. Nadia attributed overcoming these challenges to building her confidence: “... *just being more confident in holding him in the right position*”*.*

Self-efficacy was influenced by the woman’s own confidence and determination in her ability to breastfeed, and this was influenced by being in a comfortable environment, and by how much midwives and other sources of support fostered their self-efficacy. This connection is explored in the second theme – support.

### Support

This study found provision of support to be a key component of successful breastfeeding following early discharge. The sources of support participants found beneficial included their social network from partners, family and close friends, semi-professional supports such as postnatal doulas, and professional supports including midwives, nurses and lactation consultants.

Kate found simply receiving messages of encouragement from her husband who had been deployed overseas 4 days after birth as helpful: “*My husband was messaging me, telling me to do it*... *to keep going*”. Over the first few days at home Diane experienced significant breastfeeding challenges, including attachment and nipple trauma, and her baby was diagnosed with a tongue tie. Feeling emotionally and physically drained, Diane’s husband was her biggest support, especially overnight which she found “... *the most difficult time to deal with any pain with the breastfeeding*”. Her husband would assist with a top-up feed after a breastfeed while she focused on expressing: “*I couldn’t have kept up expressing if I was trying to do all that on my own overnight, it just would have been way too hard*”*.*

Ericka was not linked with timely postnatal follow up after her early discharge and so family support was of upmost importance. She drew on the experience of her aunty and mother-in-law to overcome mastitis. They gave her “... *all the hints, tips and advice on how to get through it*”. In contrast, Sally was the only participant who considered receiving advice from family as stressful. She said, “*The things they would tell me were the exact opposite of what I’ve been told to do* [by health professionals]. .. *I was very open to advice, but maybe not from them*”.

Other forms of breastfeeding support came in the form of semi-professional services. Betty and Josephine both positively described their experience of employing a postnatal doula to support their breastfeeding journey. Although Betty’s doula did provide some useful breastfeeding tips, the doula’s role involved a variety of household tasks that supported her and the baby, so they could focus on breastfeeding and bonding. Betty said, “*It just gives me a bit of respite. She will make sure I look after myself – like making sure I get sleep, or a shower, or food”,* and *“Having an extra set of hands makes such a difference*”. Josephine’s doula supported her feeding in a more direct manner providing “*reassurance and helping me know what was normal behaviour*”.

Professional breastfeeding support came in the form of midwives (in the hospital and in the home), community health nurses and lactation consultants. Midwives’ breastfeeding support styles, the way women were made to feel and the level of reassurance were three significant components of midwifery support described. Participants spoke of the need for midwives to tailor their approach to the needs of individual women. Some women valued having the midwife physically show them what to do with their breasts, while others felt violated and explained how it eroded their breastfeeding self-efficacy if a midwife physically touched their breasts. Ericka preferred the hands-on approach: “*The most unhelpful support would have been just telling me what to do rather than showing me*”. In contrast, Betty found some midwives to be quite “*forceful*” and “*… quite rough and they weren’t necessarily asking for my permission before … squeezing my boobs, or shoving it in bub’s face, and it just felt fairly uncomfortable for me*”*.*

Anna admitted that whilst not experiencing any significant breastfeeding challenges, she sought reassurance from a lactation consultant at 2 weeks postpartum. She said, “*I just wanted to clarify I was doing it right, and make sure that she was getting what she needed*”*.* Anna found the consultation with a lactation consultant affirming, stating “*It put my worries at ease, because we were doing perfectly fine and she was healthy*”. Christine sought advice from a lactation consultant at 1 week postpartum for perceived low supply, explaining that “*I wanted reassurance that I was doing okay, and my supply wasn’t dropping*”. After an assessment with the lactation consultant, Christine was left with the encouraging message “*No, you are doing everything really well. Just continue what you are doing*” which increased her confidence.

Uncertainty surrounding what is normal breastfeeding was unprecedented for these first-time mothers. This uncertainty was evident even for Cindy who is a registered midwife: “*It was hard to know what normal discomfort of baby attaching was and what wasn’t*”. These questions were resolved by reassurance from her private midwife who visited her at home: “*I spoke to her about it, and she checked a few things and said it was really normal, and it really all turned out absolutely fine*”. Cindy attributed much of her positive breastfeeding journey to the continuity model of care where she developed a relationship with one midwife: “*Having that prior relationship hugely benefits the breastfeeding journey*... *I didn’t feel like any question was too stupid*”*.*

Support in the form of answering questions, providing positive encouragement, and promoting self-efficacy was essential to the sustainability of the women’s breastfeeding journey. Breastfeeding sustainability will be explored further in theme three.

### Sustainability

The final theme, sustainability, explores how the accessibility of support and education was important for the participants to sustain their breastfeeding journey and attain their long-term breastfeeding goals. The women described timely access to breastfeeding support in the community as important. Access to different forms of community support over the course of her lactation experience was imperative for Nadia. Post her baby’s tongue-tie revision, she continued to use nipple shields and have attachment issues. She saw a lactation consultant at the hospital who said “*everything will be fine*”. Nadia said, “*I wasn’t satisfied with that, so I went and saw a Child and Family Health nurse in the community*”. Nadia was pleased with the breastfeeding support this nurse provided and continued to go back to see her: “*she was really good*... *we tried quite a few different holds until we found one that seemed to be working well. So, I’ve been going back to see her every week since then*... *it seemed to make a big difference*”. Having access to the community nurse for a second opinion was essential to Nadia’s breastfeeding sustainability.

Lori felt the timing of the provision of breastfeeding support to be important. Lori sought the support of a community nurse at 4 weeks postpartum, as she had not been contacted by them earlier as expected. While she felt the breastfeeding support she received from the nurse was satisfactory, she would have found it more helpful in the first few days and weeks. Delayed access negatively impacted Lori’s breastfeeding sustainability: “*I just really needed something extra*”.

Some women, particularly those under ‘continuity of care’ models of midwifery, valued having access to their midwife via phone or text for 6 weeks after birth. Christine, speaking of her continuity midwife, said, “*I felt that I could text or call her at any time of the day or night if I had a severe problem or anything*”. Josephine had a similar experience with her postnatal doula. She knew “*she was always available to chat, even at 10 or 11pm if I was worried*”. Knowing that someone would respond straight away or call the next day enhanced their self-efficacy which was important to these women’s breastfeeding sustainability.

Utilising online resources for breastfeeding support was also an important factor in sustaining breastfeeding for many of the women. Women sought practical support in the form of instructional videos on YouTube, gathered relevant information using Google and sought emotional support from online groups in Facebook. The women enjoyed the convenient access to the online resources and communities at all hours. Diane said, “*I’ve put questions up at 2am when you are trying to breastfeed, and you’ve got a free hand, and then someone replies within five minutes*”*.* Facebook support groups such as ‘*Breastfeeders in Australia*’ and a private lactation consultant’s page called ‘*The Milk Meg*’ were popular. Melanie, originally from Ireland, joined an Irish version of a breastfeeding support group whilst living in Australia. The women felt less alone and comforted by learning there were many other women in similar situations to them. Diane said, “*Knowing that someone else who is going through it really makes a difference*”. Similarly, Ericka said, “*Now that I know other women are going through as hard a time as I was*... *and just reading the other people’s opinions was really helpful and great. Social media is helpful*”*.*

The support gained from social media was both practical and emotionally supportive to their breastfeeding experience. Diane reflected, “*It makes you feel normal … you’re not a bad mum, you’re not doing anything wrong. There are so many different variations of normal and that your version is just one of them*”. Engaging with social media groups empowered some women to share their own experiences and support others to continue breastfeeding. Ericka said, *“I would read their worries and questions, and you could write on each other’s posts, and reply with helpful tips and things to help them*”*.* These factors all contributed to the sustainability of the women’s breastfeeding journey beyond early discharge.

## Discussion

This research aimed to understand how early discharge from the hospital within 24 h of a normal uncomplicated vaginal birth impacts the experience of first-time mothers to establish breastfeeding. The women in this study reflected on their breastfeeding journey describing the challenges they encountered and the enhancing or inhibitory factors which impacted their establishment of breastfeeding. Three inter-relational constructs of ‘self-efficacy’, ‘support’ and ‘sustainability’ were integral to the experience of initiation and ongoing breastfeeding establishment. These constructs were deeply embedded within the women’s stories and help us to understand the experience of establishing breastfeeding following early discharge.

Albert Bandura’s social cognitive theory [35, 36] defines self-efficacy as one’s confidence in their ability to effectively perform a particular task or achieve a specific goal. The participants described personal attributes and confidence as a major contributor to their experiences of breastfeeding following early discharge. Self-efficacy not only influences the commencement of a behaviour, but also powers the degree of effort and determination one puts into the task, and their sustainability of that behaviour in the face of adversity [36]. The participants’ self-belief in their capacity to breastfeed successfully influenced their ability to overcome challenges. Whilst some participants initially expressed doubt and a sense of feeling overwhelmed following their early discharge, their determination to breastfeed enhanced their self-efficacy. Maternal breastfeeding self–efficacy has been shown to be a significant predictor of breastfeeding duration and level [37]. It is well documented that having positive self-efficacy and a strong intention to breastfeed is integral to initiating and establishing breastfeeding [38–42]. However, these studies were not specific to early discharge. Women who are more confident in their ability to breastfeed in the early postpartum period have higher exclusive breastfeeding rates and are more likely to successfully breastfeed for longer durations [40, 43–45]. Fostering self-efficacy is therefore important for new mothers, and in particular for those women discharging early and with minimal support.

‘Performance accomplishment’ and ‘verbal persuasion’ are said to be significant factors influencing self-efficacy [36]. Improved confidence in one’s ability to achieve something occurs following repeated instances of success and positive experience and from receiving confirming verbal encouragement [35]. The majority of women expressed confidence in relation to breastfeeding at the time of their early discharge. They experienced positive performance accomplishment with their early feeds and felt happy to go home early with the expectation of receiving ongoing support once home.

A systematic review and meta-analysis of 11 eligible studies found that breastfeeding self-efficacy is indeed a modifiable psychological factor that midwives and other forms of breastfeeding support can influence to improve breastfeeding rates [39]. Similarly, a cross-sectional study of 571 women [46] found there to be six predictors which influenced breastfeeding self-efficacy. These were breastfeeding intention, social support, professional support from midwives/nurses, antenatal breastfeeding education classes, timing from birth to first breastfeed and previous breastfeeding experience [46]. The findings reported in the literature and the present study indicate that breastfeeding self-efficacy can be influenced and nurtured through support [39, 46]. This shows the inter-relational nature of self-efficacy and support and how together they improve breastfeeding sustainability.

The theme of support highlights how social, semi-professional and professional support influenced the establishment of breastfeeding following early discharge. Most women used family and friends for emotional, physical and verbal support to overcome breastfeeding challenges in the first few days after discharge, although one woman found advice from family to be unhelpful, outdated and stressful. Seeking help is a protective factor which enhances one’s resilience. Family members are a readily available source of support provided their values align with those of the mother. Two participants used a postnatal doula and all participants drew on the support of health care professionals, albeit at different times of their breastfeeding journey. For most women, they sought reassurance and encouragement, which is a form of ‘verbal persuasion’ and this support contributed positively to their self-efficacy and breastfeeding experience [35]. There were some instances where the participants found the ‘support’ unhelpful, and in these situations they sought a second opinion or someone who did provide the desired ‘verbal persuasion’. The women wanted to know what the indicators of ‘normal’ breastfeeding were and if they were feeding their babies correctly. Even the women with professional knowledge and those without significant challenges felt the need to seek validation from midwifes or lactation consultants.

The participants valued having ongoing, timely and convenient access to breastfeeding information, and those who did not have a midwifery group practice midwife or doula went to online resources such as Google, YouTube and Facebook to achieve this. With reduction in hospital length of stay, emphasis of breastfeeding support has naturally shifted from hospital-based postnatal midwives to increased demand on community-based social support. Nearly all mothers can successfully breastfeed, provided they have access to adequate information, and effective support from family and friends, the healthcare system and society at a macro level [47]. In the absence of readily available professional support, this study found that women are using the internet and social media for breastfeeding support. The women described accessing the internet for practical advice, information gathering, and emotional support and reassurance. These findings are echoed in current research literature, confirming the internet and social media as an emerging form of breastfeeding and postnatal support [48–51]. When evaluating women’s need for online information following childbirth, it has been found that 90.5% of women used the internet to seek information about themselves or their baby, with the most frequently searched topic being breastfeeding [50]. Most women found online resources to be useful but questioned the reliability of the information, and wanted health professionals to direct them to evidence-based, reliable online sources of information [50]. The frequent use of online resources suggests that the participants in the present study either had not received enough information prior to their early discharge or utilised these resources to enhance their confidence and support.

One approach which has embraced mobile technology as a medium for breastfeeding support in the context of early discharge is the development of a phone application in a Danish study [51]. The application was available for 7 days to participants discharged within 24 h of birth. The application included a database of information with a search function, automated messages, and a chat function with the postnatal ward staff [51]. The study found that parents felt their support needs were met, and that their self-efficacy was improved through building confidence in receiving support in a timely manner following early discharge. This type of application could be considered in the Australian setting, as a possible solution to addressing women’s desire for accessible evidence-based information and professional support.

It is clear the three themes are inter-relational and need to be considered collectively in midwifery practice. This should occur intentionally during pregnancy and not be left to chance. In the context of early discharge for first-time mothers, more could be done to promote self-efficacy through midwifery interventions and social support to address the breastfeeding self-efficacy predictors. One approach which has been shown to be successful is a breastfeeding education and support program, titled ‘The Milky Way’ program [52]. The program is based on the theoretical framework of self-efficacy and birth territory and has been shown to improve breastfeeding rates at 6 months [52, 53]. The Milky Way program includes three antenatal education classes and postnatal phone calls. The intervention focuses on improving modifiable factors influencing women’s breastfeeding decisions and confidence, namely breastfeeding intention, intrinsic power, self-efficacy and social support [52]. Broad roll out across Australia of breastfeeding support programs such as the Milky Way program could be beneficial in promoting breastfeeding self-efficacy. Such a program can be used in the context of early discharge because it is not related to postnatal stay and leads to promotion of breastfeeding sustainability.

In summary, women described the strong desire for confirmation that they were breastfeeding effectively and for encouragement to continue long term. Sustainability of breastfeeding optimises benefits to baby’s health and wellbeing. The new mothers valued ongoing and timely access to professional and semi-professional care for mother and baby that was individualised. Having timely access to quality midwifery support as well as ongoing support in other forms once discharged home early is of upmost importance for women. As the length of stay is reduced, women need to be supported, encouraged and assisted to feel confident that they are breastfeeding effectively. If this is not available to new mothers having early discharge then the three breastfeeding pillars identified in this study, namely self-efficacy, support and sustainability, will be compromised.

### Limitations

Due to the qualitative nature of interpretive research the findings are not generalisable. Having a variety of models of care and thus postnatal follow-up made it difficult to compare the women’s experience. However, the inclusion of all models of care became a strength of the study, as it demonstrated the diversity in postnatal care after early discharge. It is acknowledged that self-efficacy as a concept was not explicitly measured during the study, but rather interpretations were made from the experiences participants described. All of the women described high levels of self-efficacy, however it must be acknowledged that they self-nominated to participate in a study about breastfeeding. Women who did not self-nominate may have had a different level of self-efficacy. Nevertheless, it is clear that one’s sense of self-efficacy is an important contributor to sustaining breastfeeding.

## Conclusions

Promoting and supporting breastfeeding is a key responsibility of midwives. Early discharge following birth should not preclude midwifery support for women. This study provides insight into three inter-relational themes of self-efficacy, support and sustainability that were heavily embedded in the breastfeeding experiences of women following early discharge. The study found that self-efficacy influences the women’s readiness and motivation to breastfeed and be discharged home early and played a role in overcoming breastfeeding challenges. Social, semi-professional and professional supports were key in women’s experiences, and played an important role in promoting their breastfeeding self-efficacy. Having timely access to breastfeeding support and resources for education and reassurance was of great importance to the women who discharged early. Sustainability was thought to be enhanced by providing women ongoing, timely and convenient access to community-based breastfeeding supports and reliable online resources.

This study highlights the importance of effective breastfeeding support, encompassing the right balance of practical breastfeeding education both antenatally and postpartum, with emotional reassurance and the promotion of positive self-efficacy for women who are discharged home early. To sustain breastfeeding, women need accessible breastfeeding support, both face-to-face support and evidence-based online resources.

## Data Availability

Data sharing is not available due to ethical approval.

## References

[1] Infant feeding guidelines [https://www.nhmrc.gov.au/about-us/publications/infant-feeding-guidelines-information-health-workers] Accessed 6 Oct 2020.

[2] Victora CG, Bahl R, Barros AJD, França GVA, Horton S, Krasevec J (2016). Breastfeeding in the 21st century: epidemiology, mechanisms, and lifelong effect. Lancet..

[3] Rollins NC, Bhandari N, Hajeebhoy N, Horton S, Lutter CK, Martines JC (2016). Why invest, and what it will take to improve breastfeeding practices?. Lancet..

[4] The World Health Organization's infant feeding recommendation [http://www.who.int/nutrition/topics/infantfeeding_recommendation/en/] Accessed 21 Dec 2019.

[5] National Health Survey, Health Service Usage and Health Related Actions, Australia, 2014–15, Breastfeeding [http://www.abs.gov.au/ausstats/abs@.nsf/Lookup/by%20Subject/4364.0.55.002~2014-15~Main%20Features~Breastfeeding~10000] Accessed 14 May 2019.

[6] James L, Sweet L, Donnellan-Fernandez R (2017). Breastfeeding initiation and support: a literature review of what women value and the impact of early discharge. Women Birth.

[7] McLachlan H, Gold L, Forster D, Yelland J, Rayner J, Rayner S (2009). Women’s views of postnatal care in the context of the increasing pressure on postnatal beds in Australia. Women Birth..

[8] Australia’s Mothers and Babies 2016 [https://www.aihw.gov.au/reports/mothers-babies/australias-mothers-babies-2016-in-brief/contents/table-of-contents] Accessed 6 Oct 2020.

[9] Post Natal Ward [http://www.wch.sa.gov.au/services/az/divisions/wab/postnatalwd/index.html] Accessed 17 Oct 2019.

[10] WHO recommendations on postnatal care of the mother and newborn. [https://apps.who.int/iris/bitstream/handle/10665/97603/9789241506649_eng.pdf;jsessionid=C2FB379C23FBB655BDE56447B609A379?sequence=1] Accessed 6 Oct 2020. 24624481

[11] Bravo P, Uribe C, Contreras A (2011). Early postnatal hospital discharge: the consequences of reducing length of stay for women and newborns. Rev Gaucha Enferm.

[12] Fink A (2011). Early hospital discharge in maternal and newborn care. J Obstet Gynecol Neonatal Nurs.

[13] Budget cuts impact nurses and midwives [http://anmf.org.au/media-releases/entry/budget-cuts-impact-nurses-and-midwives] Accessed 21 Dec 2019.

[14] Ellberg L, Lundman B, Persson M, Hogberg U (2005). Comparison of health care utilization of postnatal programs in Sweden. J Obstet Gynecol Neonatal Nurs.

[15] Löf M, Svalenius E, Persson E (2006). Factors that influence first-time mothers’ choice and experience of early discharge. Scand J Caring Sci.

[16] Askeldottir B, Lam-De Jonge W, Edman G, Wiklund I (2013). Home care after early discharge: impact on healthy mothers and newborns. Midwifery..

[17] Palme L, Carlsson G, Mollbery M, Nystro M (2010). Breastfeeding: an existential challenge - women’s lived experiences of initiating breastfeeding within the context of early home discharge in Sweden. Int J QHW.

[18] Hjälmhult E, Lomborg K (2012). Managing the first period at home with a newborn: a grounded theory study of mothers’ experiences. Scand J Caring Sci.

[19] Henderson J, Redshaw M (2011). Midwifery factors associated with successful breastfeeding. Child Care Health Dev.

[20] Oakley L, Henderson J, Redshaw M, Quigley M (2014). The role of support and other factors in early breastfeeding cessation: an analysis of data from a maternity survey in England. BMC Pregnancy Childbirth.

[21] Cambonie G, Rey V, Sabarros S, Baum T, Fournier-Favre S, Mazurier E (2010). Early postpartum discharge and breastfeeding: an observational study from France. Pediatr Int.

[22] Cross-Barnet C, Augustyn M, Gross S, Resnik A, Paige D (2012). Long-term breastfeeding support: failing mothers in need. J Mother Child..

[23] Redshaw M, Henderson J (2012). Learning the hard way: expectations and experiences of infant feeding support. Birth..

[24] Walburg V, Goehlich M, Conquet M, Callahan S, Schölmerich A, Chabrol H (2010). Breast feeding initiation and duration: comparison of French and German mothers. Midwifery.

[25] Forster D, McLachlan H, Rayner J, Yelland J, Gold L, Rayner S (2008). The early postnatal period: exploring women's views, expectations and experiences of care using focus groups in Victoria, Australia. BMC Pregnancy Childbirth.

[26] Sjöström K, Welander S, Haines H, Andersson E, Hildingsson I (2013). Comparison of breastfeeding in rural areas of Sweden and Australia – a cohort study. Women Birth..

[27] Hildingsson I (2007). New parents’ experiences of postnatal care in Sweden. Women Birth..

[28] Bueno J, Romano M, Teruel R, Benjumea A, Palacín A, González C (2005). Early discharge from obstetrics-pediatrics at the hospital de Valme, with domiciliary follow-up. Am J Obstet Gynecol.

[29] Beake S, McCourt C, Bick D (2005). Women’s views of hospital and community-based postnatal care: the good, the bad and the indifferent. EBM..

[30] Johansson K, Aarts C, Darj E (2010). First-time parents’ experiences of home-based postnatal care in Sweden. Ups J Med Sci.

[31] Coughlan M, Cronin P, Ryan F (2013). Doing a literature review in nursing, health and social care.

[32] Waldenström U, Rudman A, Hildingsson I (2006). Intrapartum and postpartum care in Sweden: women’s opinions and risk factors for not being satisfied. Acta Obstet Gynecol Scand.

[33] Thorne S, Kirkham S, MacDonald-Emes J (1997). Interpretive description: a noncategorical qualitative alternative for developing nursing knowledge. Res Nurs Health.

[34] Braun V, Clarke V, Cooper H, Camic PM, Long DL, Panter AT, Rindskopf D, Sher KJ (2012). Thematic analysis. APA Handbook of Research Methods in Psychology, Vol 2: Research designs: Quantitative, qualitative, neuropsychological, and biological.

[35] Bandura A (1977). Self-efficacy: toward a unifying theory of behavioral change. Psychol Rev.

[36] Bandura A (1986). Social foundations of thought and action: a social cognitive theory.

[37] Blyth R, Creedy D, Dennis C, Moyle W, Pratt J, De Vries S (2002). Effect of maternal confidence on breastfeeding duration: an application of breastfeeding self-efficacy theory. Birth..

[38] Tuthill E, McGrath J, Graber M, Cusson R, Young S (2016). Breastfeeding self-efficacy: a critical review of available instruments. J Hum Lact.

[39] Brockway M, Benzies K, Hayden K (2017). Interventions to improve breastfeeding self-efficacy and resultant breastfeeding rates: a systematic review and meta analysis. J Hum Lact.

[40] Gerhardsson E, Mattsson E, Hildingsson I (2014). The Swedish version of the breastfeeding self-efficacy scale–short form: reliability and validity assessment. J Hum Lact.

[41] Dennis C (1999). Theoretical underpinnings of breastfeeding confidence: a self-efficacy framework. J Hum Lact.

[42] Meedya S, Fahy K, Kable A (2010). Factors that positively influence breastfeeding duration to 6 months: a literature review. Women Birth..

[43] Hinic K (2016). Predictors of breastfeeding confidence in the early postpartum period. J Obstet Gynecol Neonatal Nurs.

[44] Dennis C (2003). The breastfeeding self-efficacy scale: psychometric assessment of the short form. J Obstet Gynecol Neonatal Nurs.

[45] Bosnjak A, Rumboldt M, Stanojevic M, Dennis C (2012). Psychometric assessment of the croatian version of the breastfeeding self-efficacy scale-short form. J Hum Lact.

[46] Yang X, Gao L, Ip W, Chan W (2016). Predictors of breast feeding self-efficacy in the immediate postpartum period: a cross-sectional study. Midwifery..

[47] Breastfeeding [http://www.who.int/topics/breastfeeding/en/] Accessed 9 Nov 2019.

[48] Pettigrew S, Archer C, Harrigan P (2015). A thematic analysis of mothers’ motivations for blogging. J Mother Child.

[49] Niela-Vilén H, Axelin A, Salantera S, Melender H (2014). Internet-based peer support for parents: a systematic integrative review. Int J Nurs Stud.

[50] Slomian J, Bruyère O, Reginster J, Emonts P (2017). The internet as a source of information used by women after childbirth to meet their need for information: a web-based survey. Midwifery..

[51] Danbjørg D, Wagner L, Kristensen B, Clemensen J (2015). Intervention among new parents followed up by an interview study exploring their experiences of telemedicine after early postnatal discharge. Midwifery..

[52] Meedya S, Fahy K, Parratt J, Yoxall J (2015). Supporting women to achieve breastfeeding to six months postpartum - the theoretical foundations of a successful program. Women Birth..

[53] Meedya S, Fahy K, Parratt J (2016). The milky way educational and support programme: structure, content and strategies. Women Birth..

